# Hangboard training in advanced climbers: A randomized controlled trial

**DOI:** 10.1038/s41598-021-92898-2

**Published:** 2021-06-29

**Authors:** Saskia Mundry, Gino Steinmetz, Elizabeth J. Atkinson, Arndt F. Schilling, Volker R. Schöffl, Dominik Saul

**Affiliations:** 1 Department of Trauma, Orthopedics and Reconstructive Surgery, Georg-August-University of Goettingen, Robert-Koch-Straße 40, 37075 Göttingen, Germany; 2 Department of Quantitative Health Sciences, Clinical Trials and Biostatistics, Mayo Clinic, Rochester, MN USA; 3 Department of Trauma Surgery, Friedrich Alexander University Erlangen-Nuremberg, Erlangen, Germany; 4 Section Sports Orthopedics and Sports Medicine, Department of Orthopedic and Trauma Surgery, Klinikum Bamberg, Bamberg, Germany; 5 Kogod Center on Aging and Division of Endocrinology, Mayo Clinic, Rochester, MN 55905 USA

**Keywords:** Physiology, Health care

## Abstract

Improving climbing performance strongly depends upon effective training methods. Hangboard training is one of the most popular approaches to increase finger and forearm strength. Training protocols are based on maximizing weight or minimizing edges. We aimed to evaluate which of these protocols was superior. We prospectively analyzed 30 intermediate to advanced climbing athletes [Union Internationale des Associations d'Alpinisme (UIAA) VI–VIII] and randomized them into three groups: control group C (Control, normal climbing training), hangboard group HE (Hang endurance, grips to hold for a determined time decreased every week), and hangboard group HW (Hang weight, + 1.25 kg weight were added each week to hold for a determined time). As endpoints, we measured the grip strength before and after an 8-week training protocol in seven different pinches. Over the 8-week training period, HW hangboard training significantly improved the climbers’ grip strength compared to C [*p* = 0.032, effect size (ES) 0.36]. Maximizing weight improved the strength in I/II + III, I/II + III + IV and fist significantly. HW was superior compared to C in terms of grip strength improvement in three out of seven pinches and overall grip strength. The overall changes in the HE group did not differ significantly from the C group. An 8-week training protocol with increasing weights (HW) significantly improved overall grip strength more than a regular climbing training without the use of a hangboard.

## Introduction

Climbing sports have evolved from a niche of highly dedicated cragsman who formed the outdoor era of mountaineering into a widespread recreational and professional athletic discipline^1–3^. The rise in popularity will culminate at the Olympics in Tokyo when lead climbing, bouldering and speed climbing in a “combined mode” will have their Olympic debut on the international stage^4^. Scientific research on the optimization of performance and the validation of training schedules has not kept pace with this rapid development^5^.

Climbing performance has been associated with forearm flexor strength and endurance as well as postural control^1,6–8^. Improving forearm flexor strength as well as finger strength becomes especially important when the route characteristics become more challenging. With increasing difficulty, the grips become smaller or the distance between handholds bigger. Sometimes the negative incline grows with increasing grade of difficulty. For professional athletes, small edges and tiny pockets for one finger are not the only challenge, but frequently encountered^5^. Aside from climbing itself, specific strength training could be performed on a campusboard (a board specifically designed for vertically ascending or descending exclusively by use of the hands) or a hangboard (a board with different sizes of pockets and edges to hang on).

Even though hangboard is one of the most extensively practiced methods to enhance strong forearms and finger flexors in climbers, it has not been profoundly investigated. Diverse training protocols for hangboard training have been proposed^5,9,10^, and each of them increases grip strength and endurance by either increasing hang time or adding weight. Naturally, there is no “one fits all” protocol that helps to ameliorate every climber’s performance, as each climber has his or her own deficits and strengths^11^. We recently demonstrated that grip strength improvements are an important and early step in climbing careers^12^, but the question how to enhance grip strength is yet to be scientifically explored.

Ultimately, scientific evidence on how to improve finger grip strength and endurance after training on a hangboard/fingerboard is scarce. Regarding the upcoming and ongoing popularity of climbing sports, we aimed to compare two different training protocols, “maximizing weight” (HW) and “minimizing edges” (HE). Here we planned to evaluate the effectiveness of two hangboard training protocols over an 8-week training protocol based on maximizing weight or minimizing edges on both hands. Our primary hypothesis was that the coordinated hangboard training in the HW protocol would outweigh the improvements of the control group. In addition, we hypothesized that training in the HE group improved grip strength more compared to the C group.

## Results

### Characteristics of climbers

Out of the 30 participants, 27 completed the study (C: n = 10, HW: n = 8; HE: n = 9). Three participants did not appear at the final follow-up. The mean age of the cohort was 24.7 (± 3.5) years, and the mean body mass index (BMI) was 21.9 (± 1.4). Fifteen male (56%) and 12 female (44%) climbers participated. The mean redpoint IRCRA grade was 14 (± 4) (UIAA: 7 ± 1), while the mean on-sight IRCRA was 12 (± 3) (UIAA: 6 ± 1). Among the groups, no significant differences regarding the abovementioned parameters or grip strengths before the training protocol were observed (*p* > 0.05, one-way ANOVA. See Suppl. Tab. 3 for further sample characteristics and Suppl. Tab. 4 for pre training grip strength comparisons).

#### Between group differences—main effects model

**Figure 1:**
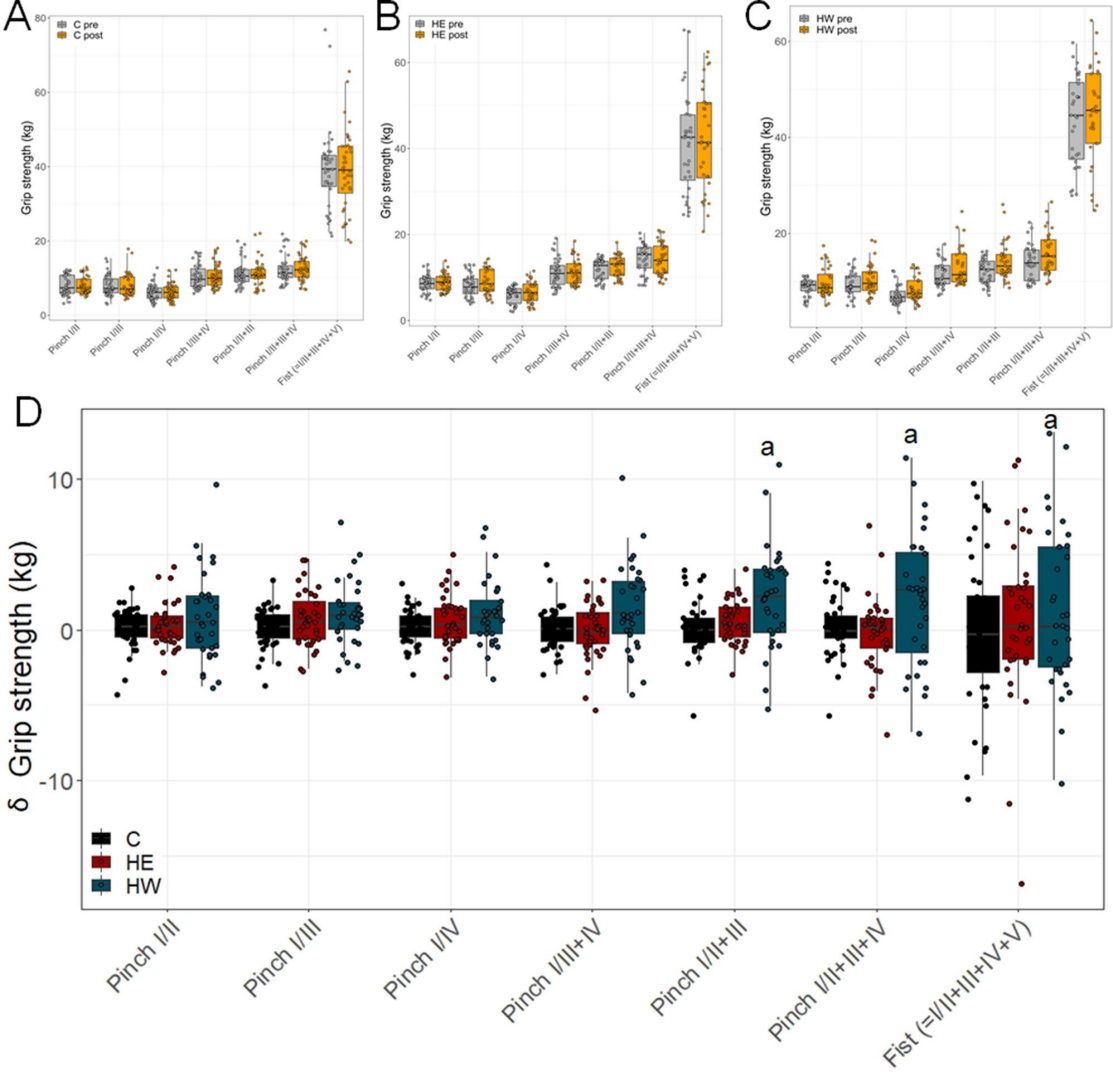
Grip strength post–pre training for each group individually and differences among groups. (**A**) In the C group. (**B**) HE group, and (**C**) HW group, the pairwise comparisons for each pinch are depicted. (**D**) A comparison between the groups showed that the HW group had improved pinch strength in I/II + III, I/II + III + IV and fist (I/II + III + IV + V) compared to the control (C) group. *a* differed significantly from C.

First, we measured the pre- and post-training grip strengths for the control group (C), HE group and HW group (Fig. 1A–C). Then, we aimed to assess whether the change from pre- to post training grip strength in either the HW or HE group differed from the change in the C group. For a comparison HW versus HE, the power of our study was not sufficient. To determine this, we created a main effects model. Here, we detected a significant difference from the C group for the HW group (*p* = 0.032, ES = 0.36), but not for the HE group (*p* = 0.417, ES = 0.15). Handedness did not significantly influence the results (*p* = 0.481) (Table 1).

**Table 1:** Mixed effects model with all 7 measurements sites.

	Estimate	Std. error	df	t value	*p* value
(Intercept)	1.513	0.567	49.4	2.67	0.010
Pre	− 0.204	0.023	737.5	− 8.70	< 0.001
Non-dom	− 0.122	0.173	732.1	− 0.71	0.481
pinch.I.III	0.218	0.318	728.5	0.69	0.492
pinch.I.IV	− 0.198	0.321	730.6	− 0.62	0.538
pinch.I.IIIandIV	0.592	0.324	731.8	1.83	0.068
pinch.I.IIandIII	1.139	0.327	733.5	3.48	0.001
pinch.I.II.and.III.and.IV	1.198	0.339	739.1	3.53	< 0.001
Fist	6.861	0.829	747.5	8.28	< 0.001
HE versus C	0.577	0.701	26.5	0.82	0.417
HW versus C	1.641	0.724	26.6	2.27	0.032

#### Between group differences—interaction site versus group

Next, we elucidated the differences among groups. Therefore, an interaction model (group vs. pinch) was designed. For every site, the comparison between HW and C was larger than between HE and C, and most of the grip strength differences in the HW group were statistically significant or almost significant. Namely, the HW group reached significantly higher grip strength differences (compared to the C group) in the first (*p* = 0.012), pinch I/II + III + IV (*p* = 0.021), and pinch I/II + III (*p* = 0.015), while the pinch I/III (*p* = 0.171) and pinch I/III + IV (*p* = 0.068) showed a trend towards improvement in the HW group. Interestingly, the HE group did not reach significant improvements in any pinch, but small effect sizes in three pinches (Tables 2 and 3, Fig. 1D). The corresponding effect sizes are listed in Table 3.

**Table 2:** Interaction group versus pinch.

Site	Group	Estimate	Std. error	df	t value	*p* value
fist	HE versus C	1.413	0.859	58.9	1.65	0.105
fist	HW versus C	2.306	0.891	60.0	2.59	0.012
pinch.I.II	HE versus C	0.408	0.858	58.6	0.48	0.636
pinch.I.II	HW versus C	0.909	0.885	58.6	1.03	0.309
pinch.I.II.and.III.and.IV	HE versus C	0.105	0.859	58.9	0.12	0.904
pinch.I.II.and.III.and.IV	HW versus C	2.109	0.886	58.7	2.38	0.021
pinch.I.III	HE versus C	0.775	0.857	58.5	0.90	0.370
pinch.I.III	HW versus C	1.229	0.886	58.7	1.39	0.171
pinch.I.IIIandIV	HE versus C	0.178	0.858	58.6	0.21	0.836
pinch.I.IIIandIV	HW versus C	1.646	0.886	58.6	1.86	0.068
pinch.I.IIandIII	HE versus C	0.659	0.858	58.6	0.77	0.446
pinch.I.IIandIII	HW versus C	2.213	0.886	58.6	2.50	0.015
pinch.I.IV	HE versus C	0.512	0.857	58.5	0.60	0.553
pinch.I.IV	HW versus C	1.091	0.886	58.6	1.23	0.223

**Table 3:** Effect size in all groups for the pre- and posttest result comparisons.

Pinch	Group 1 (HW)		Group 2 (HE)		Group 3 (C)	
	Effect size		Effect size		Effect size	
Pinch I/II	0.30	Small	0.20	Trivial	0.03	Trivial
Pinch I/III	0.31	Small	0.28	Small	0.03	Trivial
Pinch I/IV	0.39	Small	0.30	Small	0.08	Trivial
Pinch I/III + IV	0.39	Small	0.02	Trivial	0.01	Trivial
Pinch I/II + III	0.59	Medium	0.24	Small	0.03	Trivial
Pinch I/II + III + IV	0.46	Small	− 0.04	Trivial	0.04	Trivial
Fist (= I/II + III + IV + V)	0.11	Trivial	0.07	Trivial	− 0.01	Trivial

Given the high variability of the differences for the fist, we next created a model with interaction pinch versus group, but without the fist. (Suppl. Tab. 5). In this model, the differences for the HW versus the C group were more pronounced, and the pinch I/III + IV was even significantly improved compared to the C group (*p* = 0.029). All other trends mirrored the previous model with the fist included.

#### Improvements per group

**Figure 2:**
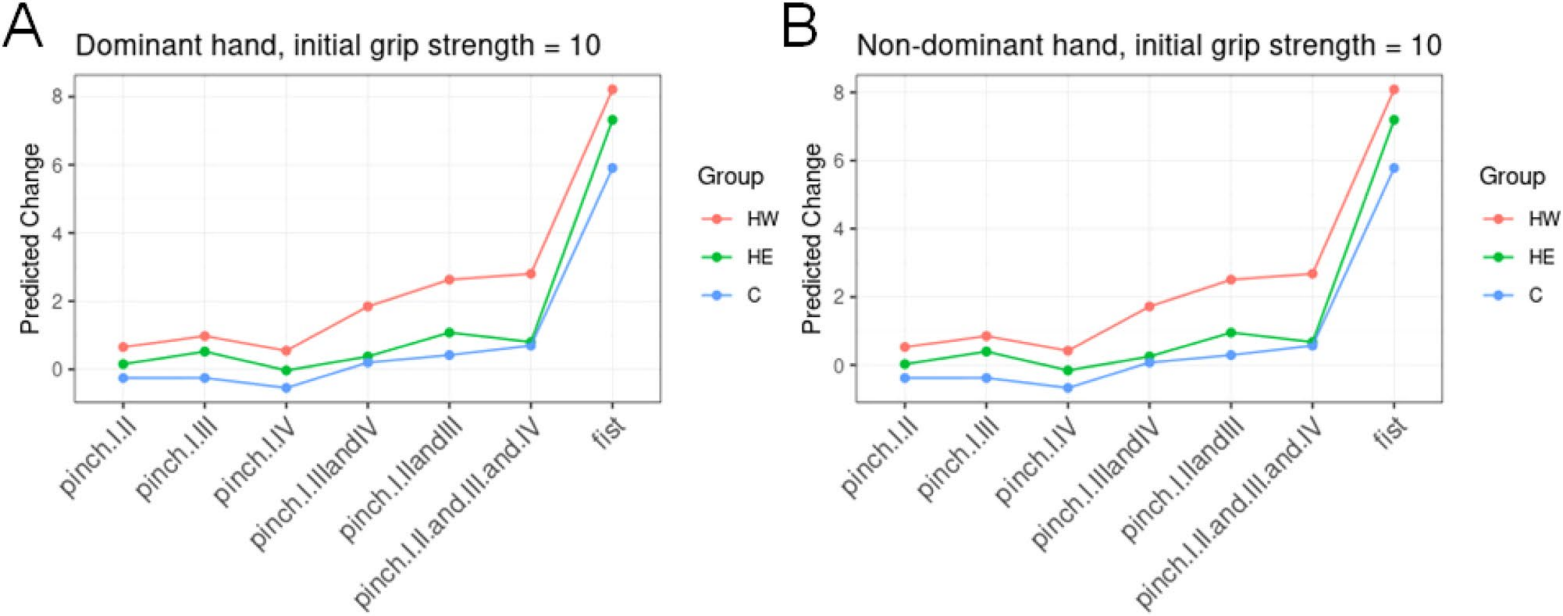
Expected grip strength improvement for each training group. For an initial grip strength of 10 kg in the pinch I/II, the predicted values for each pinch and all three groups are represented within the (**A**) dominant and (**B**) non-dominant hand.

In order to characterize the improvements within each group, we predicted the reached grip strength per pinch after an 8-week training protocol. Assuming an initial grip strength of 10 kg in the dominant and non-dominant hand, the reached grip strength per site is illustrated in Fig. 2.

## Discussion

We aimed to assess the effects of two types of 8-week hangboard training protocols and a control program regarding grip strength improvements. We reported that differences between grip strength measurements in both hands are evident after 8 weeks of hangboard training, with a superior effect of the maximizing weight protocol and an inferior effect of the minimizing edges protocol, which failed to reach significance. The improvement of almost all pinches in the HW group could arise from the fact that the measured effect after the maximizing weight protocol is getting bigger, if more than two fingers are involved. The training effect of added weights might be easier to detect the more muscles are involved, which leads to significant effects especially if more than two fingers are involved in the measurement. Subsequently, an overall effect can also be seen. In our study, a significant effect on the fist has been detected in the HW group. An explanation could be that the effects of added weight on small muscles accumulate when multiple fingers are used. The added resistance and same grip that is used over time in the HW group may lead to an increase in maximal strength, which can be captured superiorly by the dynamometer test we used in this study.

Minimizing edges, and changed grips, on the other hand, preferably affects the fist, while the overall effect is smaller than in HW. The preferential training of small flexor muscles by reducing the edge size may be measurable, but does not reach significance, although the pinches I/III, I/IV and I/II + III show small effect sizes.

The greater effect of the HW compared to the HE training protocol might result from a recruitment of more (and/or greater) muscles when weights are added. The HW training mirrors a climber-specific resistance training, focused on finger flexors. The strong effects on combined muscle activation indicate a preferential muscular hypertrophy in small flexors, especially in combination. Interestingly, both high resistance (with few repetitions) and low resistance (with high repetitions) training did improve climbers’ strength in a 10-week training program by Hermans et al.^13^. The individual pinch measurement of our study might be able to discover more profound differences at least in the HW training protocol. In addition, the participants in the Hermans study were less experienced (IRCRA 8–13 vs. IRCRA 14 (± 4)), which might have caused the lack of difference between low and high repetitions in their study^13^.

Compared to this, a smaller amount of muscles, but more specific training of these, might be needed to hold smaller edges in the HE protocol. A less resistance-focused training like HE did not improve the smaller pinches significantly (I/III, I/IV and I/II + III just insignificantly improved with small effect sizes), but the effects of HW were substantial. The fact that we tested grip strength instead of grip endurance might have missed certain effects more pronounced in the HE group, since a higher training intensity on more distal joints (distal interphalangeal joints) was created. The relatively low specificity of grip strength performance testing might also have affected the small effect sizes measured.

We found that only HW improved grip strength in the athletes over time in a statistically significant manner. While the improvements in the HW group involved almost all grips, the HE training failed to do so in the pinches I/II, I/III + IV, I/II + III + IV and fist. Only the increases in grip strength in I/II + III, I/II + III + IV and fist, and just in the HW group, were large enough to surpass the C group significantly.

Since studies comparing hangboard protocols are scarce, the studies by López-Rivera and González-Badillo^5,14^, and Medernach^15^ served as the main comparators to our findings. López-Rivera and González-Badillo performed one study with nine rock climbers in which 8 weeks of hangboard training was undertaken^14^. Different from our study, magnesium carbonate was used to reduce the perspiration effect, and the participant initially held the edge for 15–20 s. A minimum edge depth training protocol (8 to 2 mm, each training 1–2 mm deeper than the training before) was followed by a maximum added weight protocol in group A (50–90% of the previous session’s added weight), whereas group B followed the protocol in a reversed order. Measurements were performed while hanging on a 15-mm edge with an added weight (until failure of grip strength) or a smaller 11 mm edge without weight for endurance testing. The protocol that initially increased the maximum weight for 4 weeks, then minimized the edge depth for 4 weeks, appeared to be most effective in improving grip strength and endurance. Compared to that protocol, in our study, we analyzed absolute values of grip strength while the elbow was flexed. Moreover, in our study, dead hangs were not performed since endurance was not measured. However, the effect size in that study was found to be between − 0.8 and 0.7, while our values varied between 0.02 and 0.59, and especially with HW, the effects were mostly small and medium. We conclude that an 8-week training with maximum weight (HW), as we performed it, leads to a comparable effect as a 4-week minimum edge/4-week added weight training. The mean climbing ability in our group was much lower than that reported by López-Rivera and González-Badillo (UIAA VII vs. X−/X)^14^, possibly indicating a higher potential for improvement in our group.

Medernach et al. investigated 23 “highly advanced” (UIAA IX-) boulderers with two training protocols lasting 4 weeks^15^. Fingerboard and bouldering training were compared. Grip strength was assessed with a Smedley Spring Dynamometer (Saehan; Gyeonggi-do, KR), and training was performed three times a week. The protocol tested was similar to that for our fist (Pinch I/II + III + IV + V) measurements and performed only on the dominant hand. While the participants in their study were experienced in hangboard training, we chose only hangboard-naïve climbers. In their hangboard group, the fist grip strength improved from 50.2 (± 4) to 52.7 (± 4) kg, indicating a significant difference. In our HW group, improvement from 43.46 (± 9.24) to 44.73 (± 10.02) was observed, which was also significantly better compared to the C group (*p* = 0.012). Our starting level was lower, most likely due to the climbing status of recreational, not “highly advanced.” Unfortunately, Medernach et al. did not mention the effect size in their study^15^.

In their latest report, López-Rivera and González-Badillo compared three hangboard strength and endurance training programs. Advanced sport climbers (UIAA IX+/X−), who were at a higher level than our athletes (UIAA VII), participated. There, dead hangs were assessed with maximal weight until failure. The three groups performed maximum dead hangs (MaxHangs; maximum weight and minimum edge: 3–5 sets of 10 s dead hangs with 3 min rest between sets), intermittent dead hangs (IntHangs; 3–5 sets of four 10 s dead hangs, 5 s rest between repetitions and 1 min rest between sets) or 4 weeks maximum hangs followed by 4 weeks of intermittent dead hangs (Max_IntHangs). In their study, grip endurance was measured. MaxHangs and IntHangs revealed significantly enhanced endurance after 8 weeks. The effect size of their training was estimated to be between 0.2 and 1, which is comparable to our grip strength improvements, but the effect on endurance and grip force cannot be compared. Since no absolute grip strength had been assessed, only a general comparison was possible^5^.

## Limitations

Our study has several limitations. The climbing level was intermediate to advanced, and subsequently, the improvement potential cannot be transferred to elite climbers. The assessment of BMI would benefit from an additional body fat acquisition (Jackson/Pollock or Durnin’s) which will be part of future studies^16^. In addition, more detailed finger specific tests (like 3 times of 5 s contraction, followed by a certain period of prolonged rest before endurance specific tests) showed a better correlation to climbing performance than our simplified strength test protocol^17^.

The assessed groups were small (n = 27 participants in the end), and the study lacks an assessment of grip endurance (such as dead hangs) and performance testing (like test routes). The latter would have caused a longer testing period, which can cause fatigue effects but should be considered in similar future designs. Additionally, the grip dynamometry used here is a simplified but valid representative of real climbing movements, but forearm muscle activation during rock climbing differs from activation during dynamometry.

## Future directions and conclusions

Compared to a control group, maximizing weight in hangboard training with an 8-week protocol is superior in terms of grip strength advancement in recreational climbers. In the future, our training protocols will be improved, by experimentally combining the preferential effects of HW and implementing a more endurance-specific training. Additive hang endurance measurements could be conducted in order to clarify which of these protocols improves not just grip strength. Additionally, functional testing (i.e. with test routes of appropriate difficulty) should be implemented to assess climbing performance. More specific tests may allow to discriminate grip strength from hang endurance to further specify the training results. A larger group size is needed to profoundly compare more than two groups.

## Methods

### Participants

In an a priori computation of sample size (t-test, point biserial correlation model), for a power of 0.95 (estimated effect size ρ = 0.6, α = 0.05), the necessary total sample size was calculated to be n = 26 (G*Power 3.1.9.7, Kiel, Germany). Accounting for possible losses, we prospectively recruited 30 climbing athletes of an intermediate to advanced climbing level (redpoint IRCRA (International Rock Climbing Research Association 10–17 (male) or 10–14 (female) or UIAA (Union Internationale des Associations d'Alpinisme) VI–VIII (male) or VI–VII+ (female), respectively). We chose these climbing levels in order to prevent injuries in an inexperienced cohort and simultaneously since we aimed at assessing a climbing protocol for the intermediate instead of elite climbers.

Previous hangboard training experience explicitly disqualified participants for the study. Participants were randomized into three groups with the DatInf RandList program V 1.5 (Datinf GmbH Tuebingen, Germany): the control group (C), hangboard training group 1 (HE, smaller edges) and hangboard training group 2 (HW, additional weight). Basic data (age, sex) and climbing-associated data [handedness, highest climbing grade (UIAA), redpoint grade (UIAA)] were recorded. The study was approved by the institutional and licensing committee (Ethics committee of the University of Goettingen, 14/1/19) and registered at DRKS (DRKS00019838 on 11/11/2019).

All research was performed in accordance with the principles expressed in the Declaration of Helsinki, all study participants voluntarily attended the study and gave informed consent.

### Inclusion criteria

We included athletic climbers, explicitly without any previous hangboard experience. A minimum intermediate climbing level (redpoint IRCRA 10–17 (male) or 10–14 (female) or UIAA VI–VIII (male) or VI–VII+ (female), respectively) was requested.

### Exclusion criteria

Athletes younger than 18 years or with previous hangboard training experience were excluded. In addition, an acute or chronic injury on the hand or fingers or other injuries that impeded climbing were criteria for exclusion.

### Training protocols

The athletes were trained in the use of a hangboard and received instructions for the training protocols including a demonstration for one hour, before the hangboards were administered. The HE and HW groups trained two to three times a week for 8 weeks with both hands when they felt sufficiently rested (according to the proposals of López-Rivera and González-Badillo^14^). Initially, a warm-up was performed [jug (big grip) holding for 5 s, short pause, jug holding for 10 s, short pause, jug holding for 20 s, followed by as many pull-ups as possible]. In the beginning of the training protocol, on day 1 and after the warm-up, the smallest grip that could be held for 10 s was assessed and subsequently referred to as the “starting level” (SL) (level 0: Jug, level 1: 37 mm 4Fingers (F), level 2: 45 mm 3F, level 3: 20 mm 4F, level 4: 28 mm 3F, level 5: 16 mm 4F, level 6: 18 mm 3F, Suppl. Fig. 1A,B).

The following three numbers determined in each set (Suppl. Tab. 1, 2):

1. the time in seconds that a grip needs to be held (3 s in 3 × 10 × 3),
2. the length of pause in seconds (10 s in 3 × 10 × 3),
3. the number of repetitions in each set (3 repetitions in 3 × 10 × 3).

After the instructions, the participants were supervised and their progress as emerging questions monitored on a regular basis.

### Control group (C)

In this group, regular climbing training (top rope, lead and bouldering, as previously performed), on average 2.4 times (± 1.46) a week, without specially designed training on a hangboard/fingerboard (which was explicitly forbidden) was performed.

### Hangboard training group 1 (smaller edges, HE)

The objective was to increase grip strength by minimizing edges. The training protocol was performed in addition to normal climbing training on a standard retail hangboard (“Linebreaker BASE” hangboard by target10a, Ebrach, Germany, Suppl. Fig. 1A,B). Adapted and modified from MacLeod^18^, the protocol was as indicated in Suppl. Tab. 1. From week four and the first set on, the edges decreased as the starting level (SL) was continuously adjusted.

### Hangboard training group 2 (additional weight, HW)

The objective was to increase grip strength by adding weight. The training protocol was performed in addition to normal climbing training. The protocol was adapted and modified from MacLeod^18^ (Suppl. Tab. 2). From week three on, an additional + 1.25 kg of weight was added if a hold of 10 s could be accomplished in the last of the three sets.

### Diagnostic examination of grip force

All measurements were performed on both the dominant and non-dominant hands. The two hands were treated as biological, the two measurements as technical replicates.

Twenty-four hours before testing, physical activity was avoided. Grip force was measured at the same time of day with similar humidity and temperature conditions. The twice repeated measurement of maximum grip force (the output parameter was assessed in mass units of kg) was performed with a mechanograph (Leonardo Mechanograph GF, Novotec Medical GmbH, Pforzheim, Germany, Suppl. Fig. 1C). Between each measurement, one minute was granted for recovery to prevent exhaustion. For the measurements, the participants were positioned in a free position while sitting on a chair. The upper arm leant on the thorax; the elbow was 90° flexed. The hand was pronated and no compensatory movements tolerated. The validity and reliability of our measurements were assessed using Lin’s concordance correlation coefficient (CCC). A CCC of 0.99 (95% CI 0.99–0.99) showed a good concordance before and after the training protocol (Suppl. Fig. 2). In addition, we calculated the CV of our measurements. Using all the pre-treatment measurements (excluding the “fist” site) the CV was 0.24 (24%). When “fist” was included, the CV increased to 30%.

Handgrip force has been weakly correlated with actual climbing performance^17,19^. Fingers were enumerated from the thumb (I) to the little finger (V), and the “/” distinguished the two sides of the grip (i.e., a pincer grip, meaning coordination of the index finger and thumb, is referred to as “Pinch I/II”, while “Pinch I/II + III + IV” depicts the force of the thumb against the index finger + middle finger + fourth finger). The standardized order of the examined grips was Pinch I/II (mostly joints MCP I and II and IP I and PIP and DIP II), Pinch I/III (mostly joints MCP I, III and IP I and PIP and DIP III), Pinch I/IV (mostly joints MCP I and IV and IP I and PIP and DIP IV), Pinch I/III + IV (mostly joints MCP I, III and IV and IP I, PIP III and IV and DIP III and IV), Pinch I/II + III (mostly joints MCP I, II and III and IP I, PIP II and III and DIP II and III), Pinch I/II + III + IV (mostly joints MCP I- IV and IP I and PIP as DIP II-IV) and Fist (Pinch I/II + III + IV + V [mostly joints MCP I-V and IP I, PIP II-V and DIP II-V], an example is shown in Suppl. Fig. 1C).

### Statistics

To compare the numerical group characteristics (Suppl. Tab. 3 and 4), an ordinary one-way ANOVA was used after normal distribution was confirmed via D’Agostino & Pearson test. For categorial variables, a chi-square test was conducted. In all figures, the mean ± standard deviation (SD) is depicted, unless stated otherwise.

The main analysis used a mixed effects model, including the replicates and measurements from both hands. The change in grip strength (post–pre) was considered the endpoint and the covariates included the initial grip strength, measurement site (e.g., pinch.I/II, summarizing to seven measurement sites), hand (dominant/non-dominant), and treatment group. The main comparisons of interest were HE versus controls and HW versus controls. Models were fit including only main effects and including the interaction between group and measurement site. Sensitivity analyses were run fitting each grip strength site separately and the full model using only the pinch measurements (excluding the fist site). Residuals plot (fitted vs. residuals) were used to assess model fit. The replicate measurements were compared using Bland–Altman plots and summarized using the Lin’s concordance correlation coefficient (CCC) as a measure of agreement, accounting for multiple observations (sites/hand) per subject.

Effect size (g) for the change in grip strength was estimated using the Cohen’s d statistic, applying the Hedge’s correction and accounting for the paired nature of the data. According to Cohen, the effect size g˂0.2 was defined as trivial, 0.2 < g < 0.5 was defined as small, 0.5 < g < 0.8 as medium and g > 0.8 as large^20,21^. All statistical analyses were performed with SPSS Statistics 26.0 (IBM, Armonk, NY, USA) and R 4.0.3 (The R Foundation for Statistical Computing, Vienna, Austria). The graphics have been designed with R 4.0.3.

### Ethics approval

The study was approved by the institutional and licensing committee (Ethics committee of the University of Goettingen, 14/1/19) and registered at DRKS (DRKS00019838 on 11/11/2019). All research was performed in accordance with the principles expressed in the Declaration of Helsinki, all study participants voluntarily attended the study and gave informed consent.

### Consent for publication

All authors (SM, GS, EJA, AFS, VS and DS) read the final version of the manuscript and approved it.

## Supplementary information

Supplementary Informations.

## Data Availability

The raw data are available from the corresponding author upon reasonable request.
