# Supplementary material for: Hangboard training in advanced climbers: A randomized controlled trial
Source: Sci Rep. 2021 Jun 29;11:13530. doi: 10.1038/s41598-021-92898-2 (PMC8241953; doi:10.1038/s41598-021-92898-2)
Supplement: Supplementary file 1 — Supplementary Informations. [file 41598_2021_92898_MOESM1_ESM.pdf]

Hangboard training in advanced climbers – A randomized controlled trial

Mundry Saskia[1], Steinmetz Gino[1], Atkinson Elizabeth J[2], Schilling Arndt F[1], Schöffl Volker R[3,4], Saul Dominik[1,5*]

[1]Department of Trauma, Orthopedics and Reconstructive Surgery, Georg-August-University of Goettingen, Germany.

[2]Department of Quantitative Health Sciences, Clinical Trials and Biostatistics, Mayo Clinic, Rochester, MN, USA.

[3]Department of Trauma Surgery, Friedrich Alexander University Erlangen-Nuremberg, Erlangen, Germany.

[4]Section Sports Orthopedics & Sports Medicine, Department of Orthopedic and Trauma Surgery, Klinikum Bamberg, Bamberg, Germany.

[5]Kogod Center on Aging and Division of Endocrinology, Mayo Clinic, Rochester, MN 55905, USA.

*Address for correspondence

PD Dr. Dominik Saul

Georg-August University of Goettingen

Department of Trauma, Orthopedics and Reconstructive Surgery

Robert-Koch-Straße 40

37075 Goettingen, Germany

E-Mail: Dominik.Saul@med.uni-goettingen.de

Tel.: +49 551 3922462

Fax: +49 551 398787

ORCID: 0000-0002-0673-3710

Suppl. Tab. 1 Training protocol for HE (smaller edges) displaying the time in seconds that a grip needs to be held, time of pause and number of repetitions in each of the three sets (SL=Starting Level). On each training day, all three sets were performed. In the beginning of the training protocol, on day 1 and after the warm-up, the smallest grip that could be held for 10 seconds was assessed and subsequently referred to as the “starting level” (SL) (level 0: Jug, level 1: 37 mm 4Fingers (F), level 2: 45 mm 3F, level 3: 20 mm 4F, level 4: 28 mm 3F, level 5: 16 mm 4F, level 6: 18 mm 3F). For the holds, please see Fig. 4 C, D.
I.e. SL+1 means that when the starting level was 37mm 4F (level1), the new level should be 45mm 3F (level 2)

	Grip	Warm-up				
	Jug	5 sec.		10 sec.		20 sec.
	Sets					
Week 1	SL	3x10x3	SL	5x30x5	SL	5x60x3
Week 2	SL	5x10x3	SL	7x30x5	SL	7x60x3
Week 3	SL	7x60x3	SL	7-10x60x3	SL	10x60x3
Week 4	SL	7x60x3	SL+1	7-10x60x3	SL+1	10x60x3
Week 5	SL+1	7x60x3	SL+1	7-10x60x3	SL+1	10x60x3
Week 6	SL+1	7x60x3	SL+1	7-10x60x3	SL+1	10x60x3
Week 7	SL+1	7x60x3	SL+2	7-10x60x3	SL+2	10x60x3
Week 8	SL+1	7x60x3	SL+2	7-10x60x3	SL+2	10x60x3

Suppl. Tab. 2 Training protocol for HW (additional weight) indicating time in seconds that a grip needs to be held, time of pause and number of repetitions in each of the three sets (SL=Starting Level). On each training day, all three sets were performed. In the beginning of the training protocol, on day 1 and after the warm-up, the smallest grip that could be held for 10 seconds was assessed and subsequently referred to as the “starting level” (SL) (level 0: Jug, level 1: 37 mm 4Fingers (F), level 2: 45 mm 3F, level 3: 20 mm 4F, level 4: 28 mm 3F, level 5: 16 mm 4F, level 6: 18 mm 3F). For the holds, please see Fig. 4 C, D.

	Grip	Warm-up			
	Jug	5 sec.	10 sec.	20 sec.	
	Sets				
Week 1	SL	3x10x3	5x30x5	5x60x3	
Week 2	SL	5x10x3	7x30x5	7x60x3	
Week 3	SL	7x60x3	7-10x60x5	10x60x3	Warm-up and the first set (i.e. 7x60x3 for the third week) are performed. Now, a weight of 1.25 kg is added to the athlete. As soon as 10 seconds can be accomplished in this manner in the final set (i.e. 10x60x3 for the third week), another additional +1.25 kg are added until maximal additional weight is reached.
Week 4	SL	7x60x3	7-10x60x5	10x60x3	
Week 5	SL	7x60x3	7-10x60x5	10x60x3	
Week 6	SL	7x60x3	7-10x60x5	10x60x3	
Week 7	SL	7x60x3	7-10x60x5	10x60x3	
Week 8	SL	7x60x3	7-10x60x5	10x60x3	

Suppl. Tab. 3 Sample characteristics

Groups	Control (C)	Reduced grip size (HE)	Added Weight (HW)	p
N	10	9	8	
Sex (m:w)	5:5	4:5	6:2	0.4066[1]
Age [±SD]	24.0 (±3.13)	26.33 (± 4.47)	23.63 (±1.58)	0.2357[2]
BMI [±SD]	21.54 (±1.50)	22.04 (±1.38)	22.27 (±1.02)	0.5346[2]
Redpoint IRCRA-grade	12.20 (±3.25)	15.33 (±5.03)	14.50 (±3.64)	0.2728[2]
On-sight IRCRA-grade	11.00 (2.76)	13.22 (3.26)	12.28 (3.24)	0.3364[2]

Training frequency (times per week)	2.4 (±1.46)	2.72 (±1.25)	2.31 (±1.06)	0.5729[3]

[1]Chi-Square test, [2]one-way ANOVA, [3]Kruskal-Wallis

Suppl. Tab. 4 Grip strength characteristics pre training

Groups	Control (C)		Reduced grip size		Added Weight (HW)		p
	Mean (kg)	SD	Mean (kg)	SD	Mean (kg)	SD	
I/II	8.0125	2.65467	8.4778	1.98136	8.8281	1.82955	0.899[1]
I/III	7.9950	2.86105	8.0250	2.61418	9.2813	2.36962	0.259[1]
I/IV	6.1675	2.16918	5.9000	1.97846	7.1344	2.01824	0.078[1]
I/III and IV	10.4400	3.14714	11.1889	3.33619	11.5219	2.81112	0.745[1]
I/II and III	11.1225	3.37263	11.8806	2.45634	12.1125	2.55175	0.653[1]
I/II and III and IV	12.3575	3.71855	14.3667	3.40714	13.8188	3.82162	0.090[1]
Fist (I/II and III and IV and V)	39.0125	11.10861	41.1028	11.45492	43.4594	9.38320	0.952[1]

[1]one-way ANOVA

Suppl. Tab. 5: Interaction group vs. site (without fist)

site	Group	Estimate	Std. Error	df	t value	p-value
pinch.I.II	HE vs C	0.56	0.85	34.6	0.66	0.514
pinch.I.II	HW vs C	1.17	0.87	34.6	1.34	0.189
pinch.I.II.and.III.and.IV	HE vs C	0.75	0.85	35.1	0.88	0.383
pinch.I.II.and.III.and.IV	HW vs C	2.58	0.88	34.8	2.95	0.006
pinch.I.III	HE vs C	0.78	0.85	34.5	0.93	0.360
pinch.I.III	HW vs C	1.64	0.88	34.8	1.88	0.069
pinch.I.IIIandIV	HE vs C	0.42	0.85	34.6	0.50	0.623
pinch.I.IIIandIV	HW vs C	1.99	0.87	34.7	2.28	0.029
pinch.I.IIandIII	HE vs C	0.90	0.85	34.6	1.07	0.294
pinch.I.IIandIII	HW vs C	2.53	0.87	34.7	2.89	0.007
pinch.I.IV	HE vs C	0.43	0.85	34.5	0.50	0.618
pinch.I.IV	HW vs C	1.40	0.87	34.7	1.60	0.118

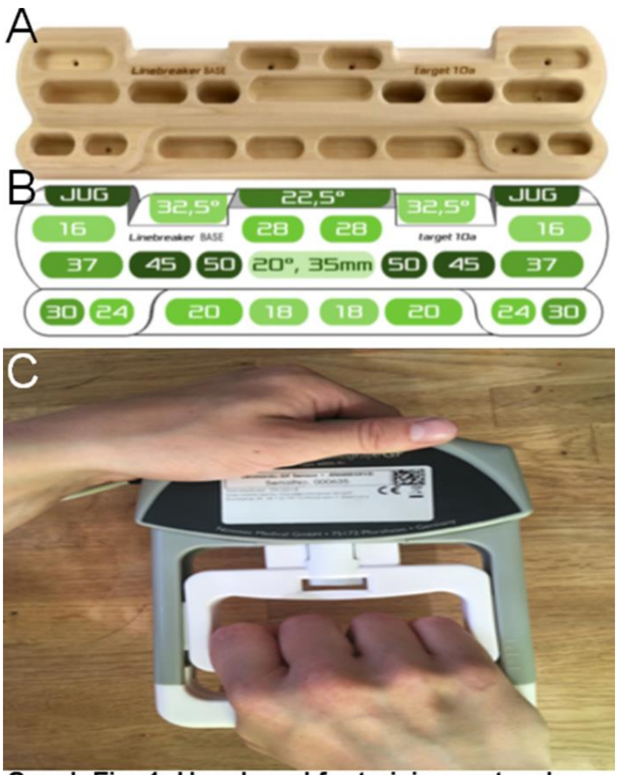

Suppl. Fig. 1: Hangboard for training protocols.
a „Linebreaker BASE" hangboard (Company „ target 10a", Ebrach, Germany). The hangboard has a difficulty around VII- to IX+ (UIAA). **b** The size of finger ledges is depicted in mm (four-finger: bars, 3-finger: bars and pockets). The image was kindly provided by target10a (Ebrach, Germany). **c** The pinch fist (=pinch I/II and III and IV and V) is exemplarily demonstrated on the Leonardo Mechanograph GF (Novotec Medical GmbH, Pforzheim, Germany).

Suppl. Fig. 1: Hangboard for training protocols. a „Linebreaker BASE" hangboard (Company „target 10a", Ebrach, Germany). The hangboard has a difficulty around VII- to IX+ (UIAA). **b** The size of finger ledges is depicted in mm (four-finger: bars, 3-finger: bars and pockets). The image was kindly provided by target10a (Ebrach, Germany). **c** The pinch fist (=pinch I/II and III and IV and V) is exemplarily demonstrated on the Leonardo Mechanograph GF (Novotec Medical GmbH, Pforzheim, Germany).

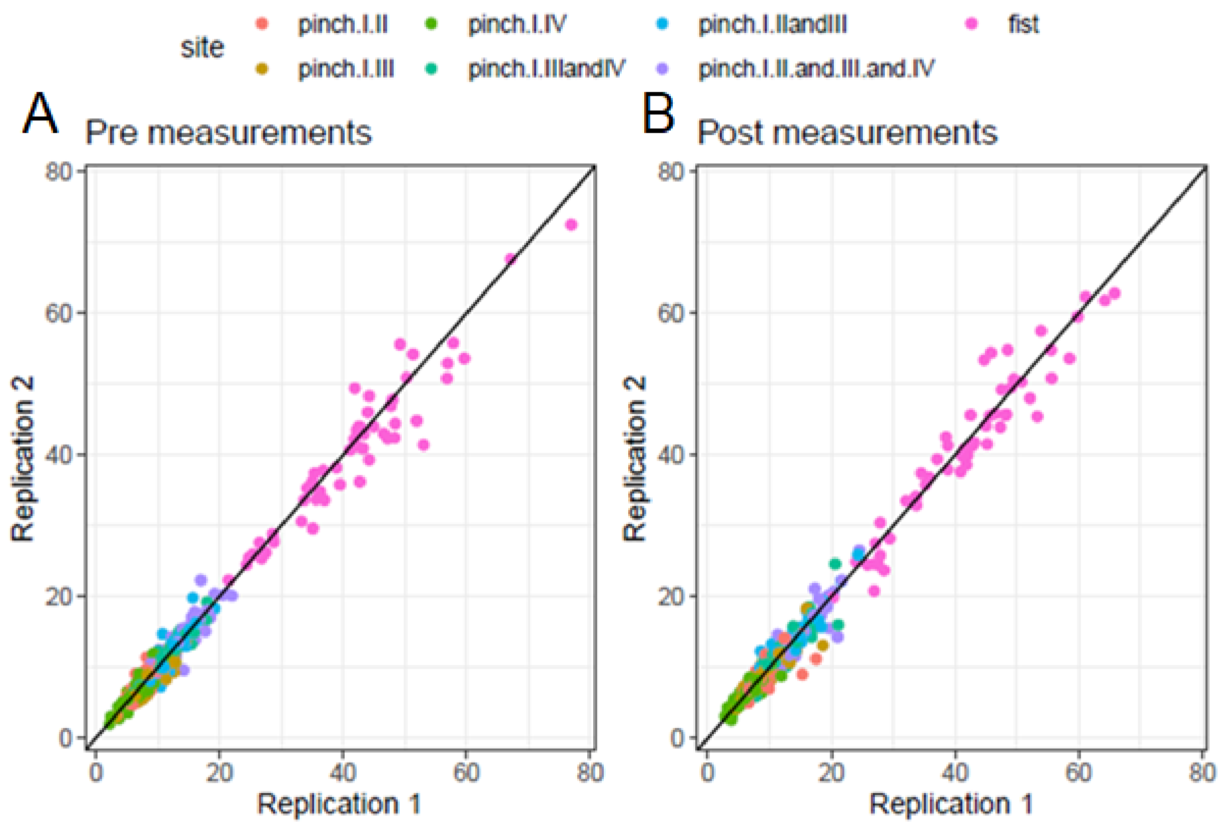

Suppl. Fig. 2: Agreement between replicative measurements. Comparing replication 1 and 2, the overall CCC was 0.99 (95%-CI 0.99-0.99), indicating a good agreement of the replicative measurements in both **a** pre and **b** post measurements.

Suppl. Fig. 2: Agreement between replicative measurements. Comparing replication 1 and 2, the overall CCC was 0.99 (95%-CI 0.99-0.99), indicating a good agreement of the replicative measurements in both **a** pre and **b** post measurements.

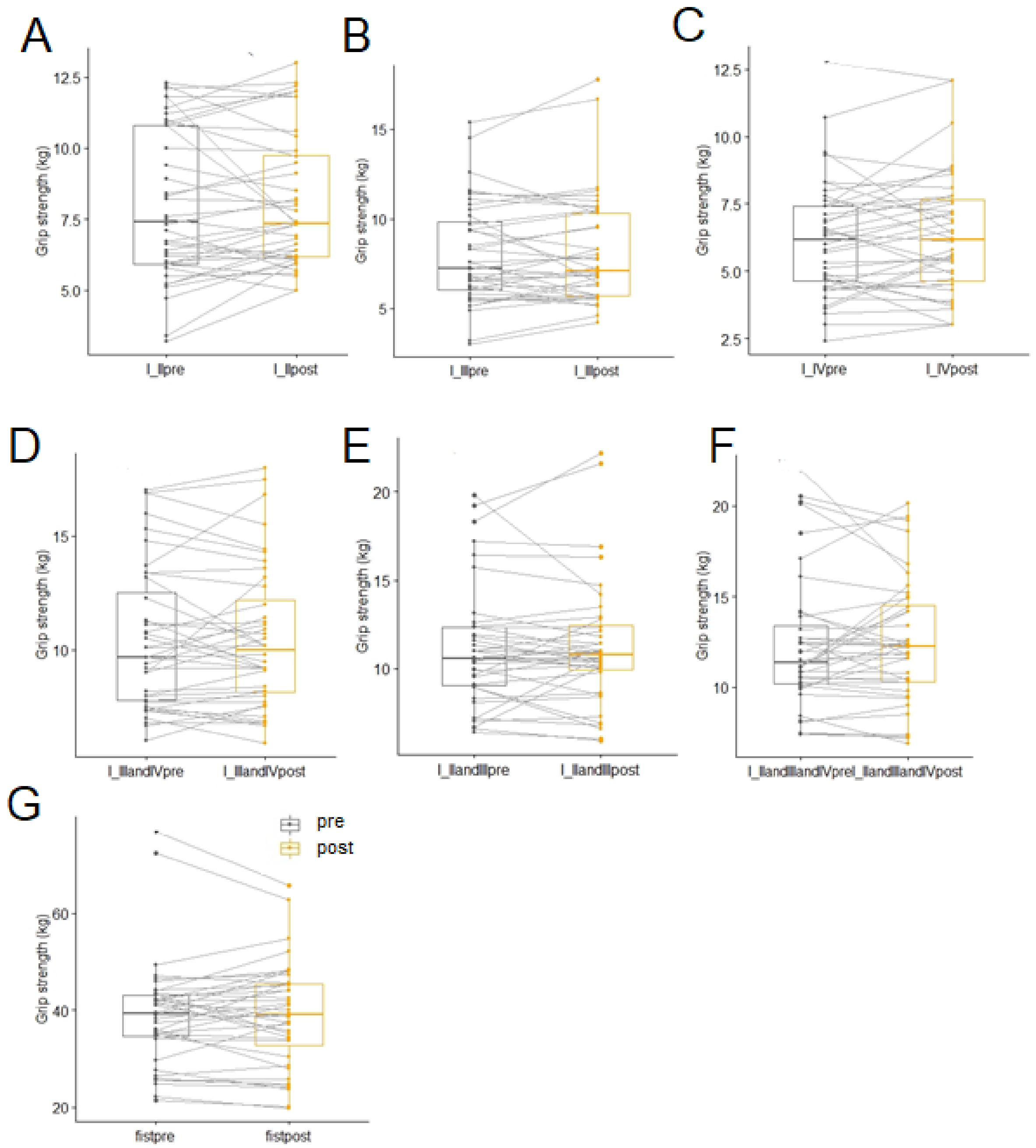

Suppl. Fig. 3: Individual changes for each pinch within the C group. The pairwise changes are depicted for **a** pinch I/II, **b** pinch I/III, **c** pinch I/IV, **d** pinch I/III+IV, **e** pinch I/II+III, **f** pinch I/II+III+IV and **g** fist.

Suppl. Fig. 3: Individual changes for each pinch within the C group. The pairwise changes are depicted for **a** pinch I/II, **b** pinch I/III, **c** pinch I/IV, **d** pinch I/III+IV, **e** pinch I/II+III, **f** pinch I/II+III+IV and **g** fist.

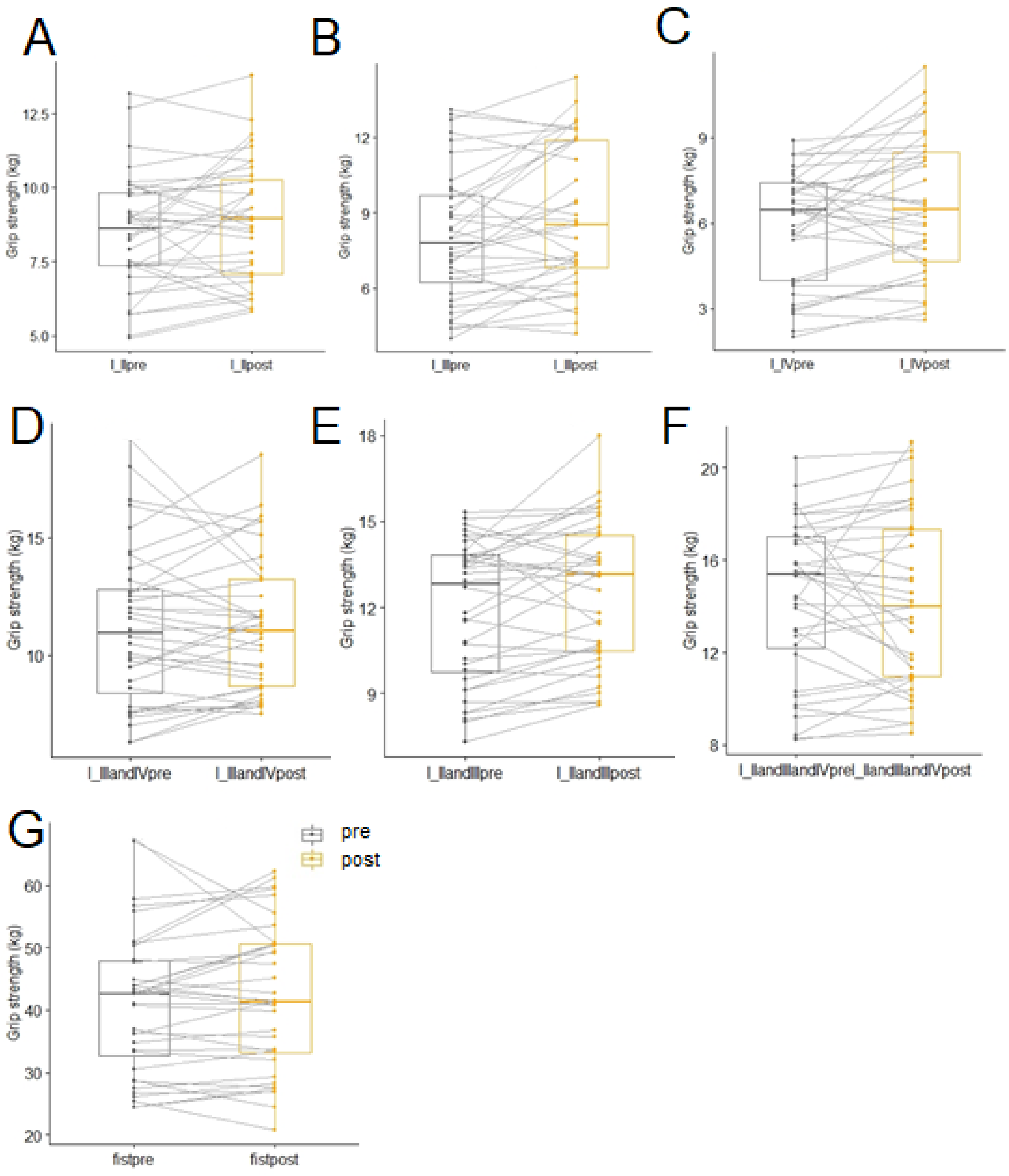

Suppl. Fig. 4: Individual changes for each pinch within the HE group. The pairwise changes are depicted for **a** pinch I/II, **b** pinch I/III, **c** pinch I/IV, **d** pinch I/III+IV, **e** pinch I/II+III, **f** pinch I/II+III+IV and **g** fist.

Suppl. Fig. 4: Individual changes for each pinch within the HE group. The pairwise changes are depicted for **a** pinch I/II, **b** pinch I/III, **c** pinch I/IV, **d** pinch I/III+IV, **e** pinch I/II+III, **f** pinch I/II+III+IV and **g** fist.

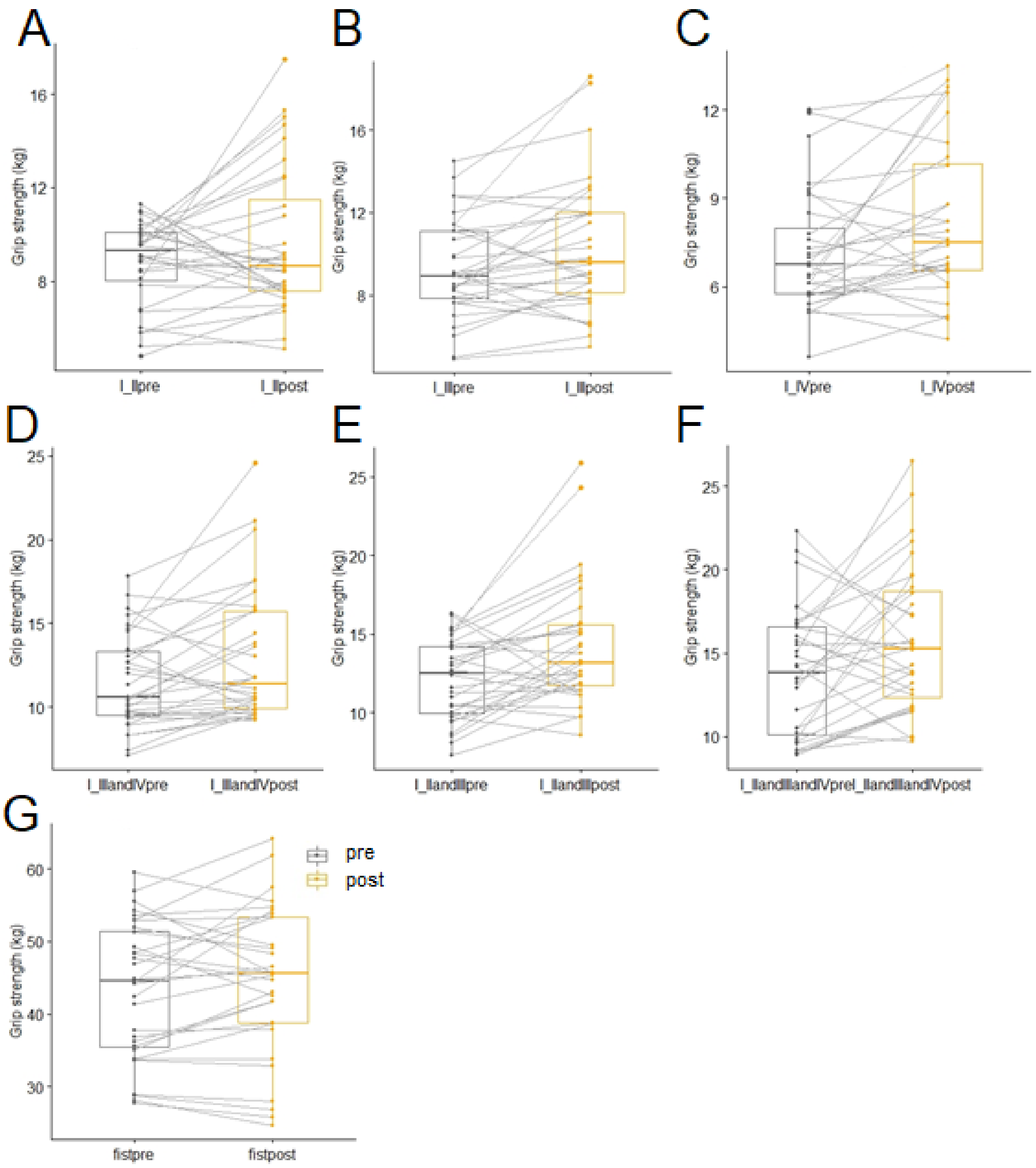

Suppl. Fig. 5: Individual changes for each pinch within the HW group. The pairwise changes are depicted for **a** pinch I/II, **b** pinch I/III, **c** pinch I/IV, **d** pinch I/III+IV, **e** pinch I/II+III, **f** pinch I/II+III+IV and **g** fist.

Suppl. Fig. 5: Individual changes for each pinch within the HW group. The pairwise changes are depicted for **a** pinch I/II, **b** pinch I/III, **c** pinch I/IV, **d** pinch I/III+IV, **e** pinch I/II+III, **f** pinch I/II+III+IV and **g** fist.%Y
